# Primary fibroblastic osteosarcoma of sternum: A case report

**DOI:** 10.1097/MD.0000000000028827

**Published:** 2022-02-18

**Authors:** Shaozi Fu, Yile Zhou, Heyun Xu

**Affiliations:** a Department of Thoracic Surgery, The First Affiliated Hospital, Zhejiang University School of Medicine, Hangzhou, China; b Department of Hematology, The First Affiliated Hospital, Zhejiang University School of Medicine, Hangzhou, China.

**Keywords:** fibroblastic osteosarcoma, reconstruction, sternum, whole-exome sequencing

## Abstract

**Rationale:**

Osteosarcoma, a malignant bone tumor, rarely occurs in the sternum, especially the fibroblastic type, and is associated with poor survival. This case report describes a patient with a neoplasm of the sternum who underwent tumor resection 3 times and reconstruction twice because of the high risk of recurrence.

**Patient Concerns:**

A 60-year-old Chinese man presented with a 3-cm palpable bulging mass located in front of his sternum. Chest computed tomography (CT) revealed an anterior chest wall neoplasm with sternal destruction.

**Diagnosis:**

Pathological examination revealed that the mass was a low-grade malignant primary fibroblastic osteosarcoma.

**Interventions:**

Locking plates were used for chest wall reconstruction, demonstrating good structural stability and economic applicability. Regarding the ineffectiveness of current therapies, whole-exome sequencing was conducted, and no targets matched any of the currently available agents.

**Outcomes:**

No recurrence was found on regular reexamination.

**Lessons:**

Surgery is the first choice of treatment for patients with primary fibroblastic osteosarcoma of the sternum. The reconstruction-locking plate is a good alternative for chest wall reconstruction. Whole-exome sequencing can shed new light on this uncommon disease and help identify novel therapeutic targets.

## 1 Introduction

Osteosarcoma is a malignant bone tumor that most frequently occurs in the extremities of children and adolescents. However, its occurrence in the chest wall, especially in the sternum, is infrequent. The average age of patients with osteosarcoma of the sternum is approximately 42 years at diagnosis.^[1]^ Osteosarcoma is histologically classified into osteoblastic (76%–80%), chondroblastic (10%–13%), fibroblastic (10%), and other rare variants.^[2]^ Therefore, primary fibroblastic osteosarcomas arising in the sternum are extremely rare. Conventional therapies for chest osteosarcoma include surgical resection, chemotherapy, and radiotherapy, which have poor survival.^[3]^ The prognostic factors include response to chemotherapy, association with Paget's disease, and unifocal osteosarcoma. This case report demonstrates the clinical presentation, treatment, and whole-exome sequencing of a primary fibroblastic osteosarcoma of the sternum.

## 2 Case presentation

**Figure 1:**
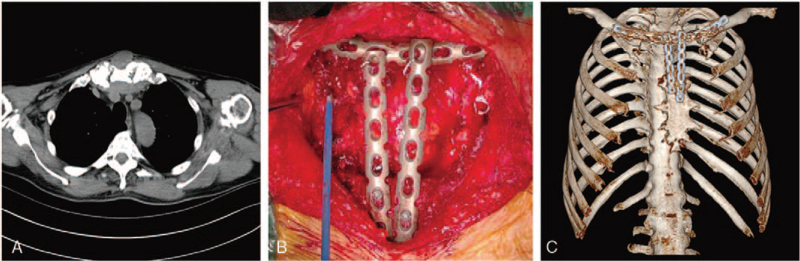
(A) Chest CT scanning displayed an anterior chest wall neoplasm. (B) The second operation of tumor resection and chest wall reconstruction. (C) CT review after the second operation.

**Figure 2:**
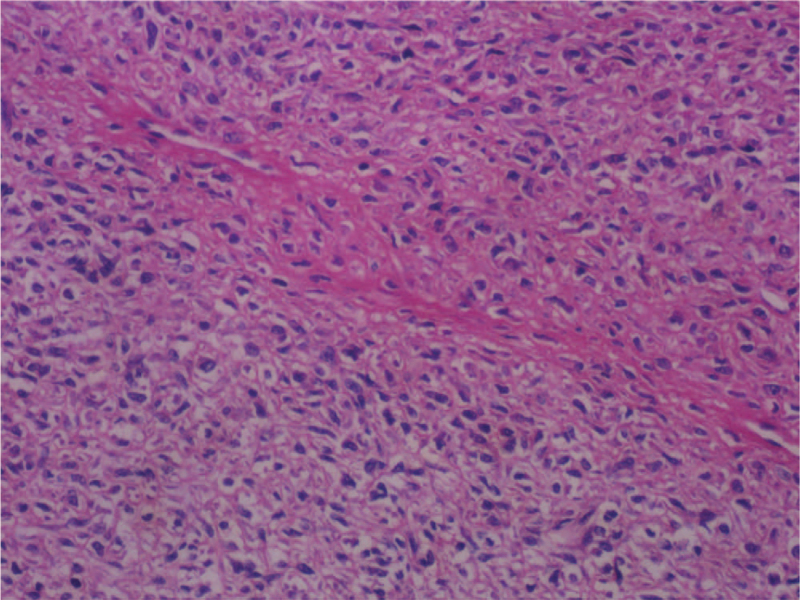
A pathological examination demonstrating fibroblastic osteosarcoma, infiltrating soft tissue (magnification, ×400).

A 60-year-old man presented with a 3-cm anterior chest neoplasm, without any positive symptoms. The mass was misdiagnosed as a benign tumor at a local hospital. Local excision was performed, and the pathological results revealed a solitary fibrous tumor. One year later, the neoplasm relapsed and the patient was referred to our department. Physical examination demonstrated a 3-cm palpable bulging mass located directly in front of the sternum. Routine blood tests, biochemical tests, tumor markers, and other laboratory examinations revealed no abnormalities. Chest computed tomography (CT) revealed an 8.5-cm anterior chest wall neoplasm with 3-cm deep infiltration of the sternum (Fig. 1A), and whole-body bone scan revealed no apparent abnormalities. According to the 8th AJCC staging system, the tumor was classified as stage IB. When preoperative pulmonary and cardiac function were evaluated normal, the wide local excision and reconstruction were performed using a “T” incision. The tumor was removed simultaneously with the manubrium and proximal third of the bilateral clavicles. Three reconstruction locking plates (CanSLP system, Canwell) were used for reconstruction, forming a pattern of “π” (Fig. 1B). The patient recovered well, and CT revealed a stable anterior chest structure (Fig. 1C). Finally, pathological examination confirmed that the chest mass was a low-grade, malignant primary fibroblastic osteosarcoma with negative margins (Fig. 2). Immunohistochemical staining demonstrated that tumor cells were positive for special AT-rich sequence-binding protein 2 (SATB2), Bcl-2, vimentin, and CD34, but negative for S-100 protein, epithelial membrane antigen (EMA), and smooth muscle actin (SMA).

**Figure 3:**
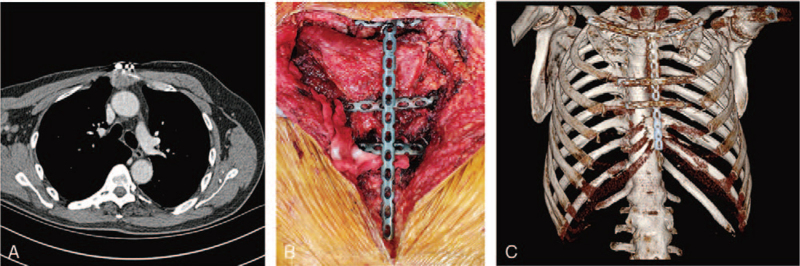
(A) Chest CT revealed tumor recurrence again. (B) The third operation of tumor resection and chest wall reconstruction. (C) CT review after the third operation.

Unfortunately, the anterior chest wall neoplasm relapsed eight months later. Chest computed tomography (CT) revealed a 3-cm neoplasm in the anterior chest wall and residual mesosternum with a 2-cm deep infiltration (Fig. 3A). The tumor was stage IA. Oncologists recommended surgical resection over chemotherapy and radiotherapy because of their poor efficacy in primary fibroblastic osteosarcoma; therefore, a reoperation was performed. We removed two-thirds of the mesosternum and bilateral third and fourth costicartilages and freed a large pectoralis myocutaneous flap to cover the defect caused by destruction. Four additional reconstruction-locking plates (CanSLP system, Canwell) were used for reconstruction, resulting in a stable structure (Fig. 3B and C).

Concerning the negative consequences, whole-exome sequencing was conducted to determine whether there are any well-known gene mutations for which we presently have target agents, with ethics committee approval and informed consent obtained from the patient. The results revealed 314 single-nucleotide variants (SNVs), including 9 synonymous mutations, without currently available targets. This detection revealed 4 potentially meaningful SNVs, including *MAGI1 (membrane-associated guanylate kinase with an inverted repeat member 1), BBS9 (Bardet-Biedl Syndrome 9)*, *Alpha-Sarcoglycan (SGCA),* and *GALK1 (Galactokinase 1)*. These 4 SNVs are missense mutations located in exons. Protein function prediction was performed using SHIF, Polyphen2, and MutationTaster, all of which were deleterious. The GERP++ scores of these genes, analyzed by dbNSFP version 3.0, were >2, indicating that these genes may be located in conserved regions.

Since the last operation, the patient was accessed every 6 months for one year and a half. The patient recovered well and was able to engage in light labor. Physical examination and CT did not reveal any recurrence.

## 3 Discussion and conclusion

Osteosarcoma typically occurs between the ages of 10–14 years and affects the long bones, including the femur, tibia, and humerus.^[4]^ Previous studies have shown that <1% of primary bone tumors occur in the sternum, and chondrosarcoma is the most prevalent type of primary malignant tumor in the sternum.^[5]^ Patients with osteosarcoma of the sternum often present with an enlarged painful neoplasm, and 34% of them might have metastasis.^[6]^

We reviewed the existing case reports of osteosarcoma of the sternum (Table 1). A total of 6 cases have been reported, and there is only one case of fibroblastic osteosarcoma reporting the same pathological result as our case.^[^1^,^^6^^–^9^]^ Five of the 6 patients underwent extensive surgical resection with or without neo-oradjuvant treatment. Only 1 case chose chemoradiotherapy because of distal metastasis. Overall, surgery is the first choice unless the patient presents with metastasis.^[7]^ Wide surgical excision of the primary neoplasm is recommended for localized disease.^[6]^

**Table 1:** A literature review on the existing case reports of the osteosarcoma of sternum.

Case	Age, y	Sex	Pathology	CT scanning	Treatment	Recurrence	Reference
1	60	Female	Fibroblastic osteosarcoma	The tumor involved the upper mediastinum and displaced the left innominate vein without infiltrating it	Surgery and adjuvant chemotherapy	Not within 16 mo	Briccoli et al, 2002^[8]^
2	36	Male	Chondroblastic osteosarcoma	A large destructive anterior mediastinal mass involving the manubrium and sternum with multiple bilateral calcified lung masses	Chemoradiotherapy	Not mentioned	Masab et al, 2018^[6]^
3	24	Male	Chondroblastic osteosarcoma	Irregular calcified mass of the soft tissue	Neoadjuvant chemotherapy and surgery	Not within 19 mo	Briccoli et al, 2002^[8]^
4	74	Male	Unspecified osteosarcoma	A calcified mass with central necrosis	Surgery and adjuvant radiation	Not within 2 y	Douglas et al, 2009^[1]^
5	71	Male	Unspecified osteosarcoma	A destructive lesion of the manubrium, with an associated soft tissue mass extending into the pectoralis muscle and anterior mediastinum	surgery	Not mentioned	Weinberg et al, 2009^[9]^
6	57	Male	Unspecified osteosarcoma	A large densely ossified mass located on the manubriosternal angle, extending posteriorly into the mediastinum and displacing the overlying anterior soft tissues	Neoadjuvant chemotherapy, surgery and adjuvant chemotherapy	Not within 1 y	Rad et al, 2014^[7]^

Herein, we report a case of primary fibroblastic osteosarcoma of the sternum. In this case, the patient presented with a superficial painless tumor, chest CT scanning indicated tissue infiltration, and whole-body bone scan showed no bone metastasis. This might be the reason why the local hospital misdiagnosed the case as an SFT. We performed reconstructions twice using locking plates rather than 3D printing materials owing to the financial constraints of patients. Fortunately, these structures were stable. This could be the ideal surgical option for patients without medical insurance.

Given the poor prognosis of this rare disease, we performed whole-exome sequencing, which has not been performed previously. The results indicated 314 SNVs, including nine synonymous mutations. Although no targets correspond to currently available agents, we provide novel perspectives and possible targets that require additional study to elucidate their functions in this disease.

## Author contributions

**Data curation:** Shaozi Fu.

**Investigation:** Yile Zhou.

**Writing – original draft:** Shaozi Fu, Yile Zhou.

**Writing – review & editing:** Heyun Xu.
